# Detailed Insight into Photocatalytic Inactivation of Pathogenic Bacteria in the Presence of Visible-Light-Active Multicomponent Photocatalysts

**DOI:** 10.3390/nano14050409

**Published:** 2024-02-23

**Authors:** Magda Kozak, Paweł Mazierski, Joanna Żebrowska, Tomasz Klimczuk, Wojciech Lisowski, Andrzej M. Żak, Piotr M. Skowron, Adriana Zaleska-Medynska

**Affiliations:** 1 Department of Environmental Technology, Faculty of Chemistry, University of Gdansk, 80-308 Gdansk, Poland; pawel.mazierski@ug.edu.pl; 2 Department of Molecular Biotechnology, Faculty of Chemistry, University of Gdansk, 80-308 Gdansk, Poland; joanna.zebrowska@ug.edu.pl (J.Ż.); piotr.skowron@ug.edu.pl (P.M.S.); 3 Faculty of Applied Physics and Mathematics, Gdansk University of Technology, 80-233 Gdansk, Poland; tomasz.klimczuk@pg.edu.pl; 4 Institute of Physical Chemistry, Polish Academy of Sciences, 01-224 Warsaw, Poland; wlisowski@ichf.edu.pl; 5 Faculty of Chemistry, Wroclaw University of Science and Technology, 50-370 Wroclaw, Poland; andrzej.zak@pwr.edu.pl

**Keywords:** heterogeneous photocatalysis, ternary alloys, semiconductors, anodization, nanomaterials fabrication, removal of microbiological pollutants

## Abstract

The use of heterogeneous photocatalysis in biologically contaminated water purification processes still requires the development of materials active in visible light, preferably in the form of thin films. Herein, we report nanotube structures made of TiO_2_/Ag_2_O/Au^0^, TiO_2_/Ag_2_O/PtO_x_, TiO_2_/Cu_2_O/Au^0^, and TiO_2_/Cu_2_O/PtO_x_ obtained via one-step anodic oxidation of the titanium-based alloys (Ti_94_Ag_5_Au_1_, Ti_94_Cu_5_Pt_1_, Ti_94_Cu_5_Au_1_, and Ti_94_Ag_5_Pt_1_) possessing high visible light activity in the inactivation process of methicillin-susceptible *S. aureus* and other pathogenic bacteria—*E. coli*, *Clostridium* sp., and *K. oxytoca*. In the samples made from Ti-based alloys, metal/metal oxide nanoparticles were formed, which were located on the surface and inside the walls of the NTs. The obtained results showed that oxygen species produced at the surface of irradiated photocatalysts and the presence of copper and silver species in the photoactive layers both contributed to the inactivation of bacteria. Photocatalytic inactivation of *E. coli*, *S. aureus*, and *Clostridium* sp. was confirmed via TEM imaging of bacterium cell destruction and the detection of CO_2_ as a result of bacteria cell mineralization for the most active sample. These results suggest that the membrane ruptures as a result of the attack of active oxygen species, and then, both the membrane and the contents are mineralized to CO_2_.

## 1. Introduction

Photocatalysts are substances that can initiate or accelerate a chemical reaction using light energy. They are widely used in various applications, including environmental remediation, hydrogen production, and organic synthesis [1,2], as well as microbial elimination [3]. While many photocatalysts are based on single-component materials such as titanium dioxide (TiO_2_), there is also ongoing research into developing more complex photocatalysts using multi-component alloys.

One of the most promising forms of nanostructures active in photocatalytic processes is TiO_2_ nanotubes (NTs). Among other factors, NTs could enable better control of chemical and physical properties. By reducing the dimensions to the nanoscale, not only does the surface area increase significantly but the electronic structure also undergoes significant changes (e.g., due to the occurrence of the quantum effect or the curvature of the surface) [4]. These NTs can be synthesized using various methods, such as hard matrix, hydro/solvothermal, electrospinning, and anodic oxidation methods, obtaining a material in the form of a powder or a thin layer [5]. Improvement of visible-light-induced photocatalytic activity of TiO_2_ NTs arrays could be obtained via doping with non-metals and metals [6], the deposition of noble metal nanoparticles [7] and quantum dots [8], the formation of semiconductor heterojunctions [9,10], or atomic layer deposition [11].

During the creation of NTs, coupling with semiconductor oxides can be accomplished quickly and directly via the anodization of alloy foils containing Ti and a modifying metal. It was confirmed that obtaining NTs via electrochemical methods from alloys combined of Ti and a second metal [12,13,14,15,16,17,18,19,20,21,22,23,24] is possible and promising in various applications.

A very interesting approach nowadays involves alloying semiconductor materials to create photocatalysts with extended light absorption, improved charge carrier dynamics, and antibacterial properties. Researchers have explored the alloying of titanium dioxide (TiO_2_) with other semiconductors like silver oxide (Ag_2_O) [13] or copper oxide (CuO) [12] to improve photocatalytic activity, extend the absorption range to the visible light region, and increase its disinfection efficiency. Alongside metal oxides, plasmonic metals like gold or platinum can strengthen the photocatalytic process and increase visible light absorption through the localized surface plasmon resonance (LSPR) effect [25]. The excited electrons and enhanced electric fields produced in the presence of the NPs can promote the photocatalytic reactions that convert solar energy into chemical energy. 

In the case of photocatalysts in the form of NTs formed from alloys that consist of more than two metals, there are not many studies published on these materials [20,21,23,24,26,27,28,29]. It was confirmed that obtaining NTs from complex alloys in the electrochemical oxidation process is possible, and moreover, new structures are active in the photocatalytic processes and influence bacterial inactivation. Ternary nanostructured photocatalysts originating from alloys offer several advantages over other photocatalysts in terms of scalability. The unique structure and composition of ternary photocatalysts allow for tunable properties, chemical stability, and reusability, which are essential for practical applications. In terms of chemical stability, ternary photocatalysts often exhibit improved chemical stability compared to their single-component counterparts. This is due to the formation of a more robust and less-prone-to-degradation material, which can lead to longer-lasting photocatalysts. The stability of photocatalysts originating from metal alloys can also contribute to their reusability as they can maintain their photocatalytic activity even after multiple cycles of use [30]. The process of scaling up in terms of the anodization method is relatively easy to perform as it only requires an increase in the area of electrodes, and there are fewer process parameters to consider compared to other methods such as in hydrothermal methods. It can also be a cost-effective method as it requires fewer steps of synthesis than the solvothermal method [31,32]. But the most important feature is the complex structure obtained during a single-stage process of formation, which is money- and time-saving.

In the literature, there are many examples of *E. coli* inactivation by various types of photocatalysts in the presence of visible light [3,33]; however, the impact of the photocatalytic process on *S. aureus*, which is a big problem in healthcare facilities and communities [34], has not been so intensively studied. Moreover, studies on the combination of TiO_2_/Ag_2_O or TiO_2_/Cu_2_O with noble metal NPs that may affect the inactivation of bacteria in visible light were not found in the literature. Therefore, the authors decided to obtain new hybrid NT layers via simple anodization of multicomponent alloys composed of 94% wt. of titanium, 5% wt. of silver or copper metal, and 1% wt. of gold or platinum used as a substrate to obtain visible-light-active—and ready for multiple use—NT layers composed of the expected TiO_2_, Ag_2_O, Ag^0^, Cu_2_O, Au^0^, PtO_x_, or Pt^0^. The impact of alloying titanium dioxide with other semiconductors like silver oxide or copper oxide has been primarily investigated on *S. aureus* (MSSA—methicillin-susceptible *Staphylococcus aureus*), and subsequently on other harmful bacteria that are important for the human environment such as *E. coli*, *Clostridium sp*., and *K. oxytoca*. Promising results hold potential for applications in various fields, including medical devices, antimicrobial coatings, and environmental remediation.

## 2. Materials and Methods

### 2.1. Chemicals and Reagents

The titanium foils and alloys Ti_x_Cu_y_M_z_ and Ti_x_Ag_y_M_z_—where M = Pt or Au; x = 94% wt., y = 5% wt., and z = 1% wt. (Ti_94_Ag_5_Au_1_, Ti_94_Cu_5_Pt_1_, Ti_94_Cu_5_Au_1_, Ti_94_Ag_5_Pt_1_)—were bought from the company HMW Hauner (Roettenbach, Germany). Acetone, isopropanol, methanol, ethylene glycol (99.0%, p.a.), and NH_4_F (p.a.) were purchased from POCh S.A. (Gliwice, Poland). The conductivity of the deionized water used in the studies was 0.05 µS. The bacterial strains used in this work were as follows: *Escherichia coli* (*E. coli*) DSMZ collection no 1116, *Clostridium* sp. DSMZ collection no 2634, *Staphylococcus aureus* (*S. aureus*) KPD 740 (MSSA), and *Klebsiella oxytoca* (*K. oxytoca*) KPD 734 (ESBL). 

### 2.2. Synthesis of Nanotubes

Ti foil and Ti alloys were cut into 2.5 cm × 2.5 cm pieces. Initially, samples were cleaned in an ultrasonic bath for 10 min with acetone, isopropanol, methanol, and deionized water, in that order. The final step of the cleaning process was air steam drying. Moreover, two electrodes were used in the anodization process: prepared alloys as the working electrode and platinum mesh as the cathode, with a distance of 2 cm between them. The electrolyte solution was composed of ethylene glycol (98 vol %), deionized water (2 vol %), and NH_4_F (0.09 M). The stirring of electrolyte was applied (500 rpm). We immersed 2 cm of Ti foil and Ti alloys in 150 mL of electrolyte. The anodic oxidation process was controlled by applying a voltage of 30 V to the working electrode (the current density decreased from 25 to 2 mA/cm^2^) for 60 to 90 min. The samples were then sonicated in deionized water for the last preparation phase, dried in an air stream at 80 °C for 24 h, and then calcined for an hour at 450 °C (heating rate of 2 °C/min). Detailed information is given in Table 1. 

### 2.3. Characterization Systems

To understand the morphology of the obtained structures, SEM and X-ray photoelectron spectroscopy were used. A PHI 5000 VersaProbeTM (Ulvac PHI, Chigasaki, Japan), spectrometer and a JOEL JSM-7610F (JOEL Ltd., Tokyo, Japan) device were employed in the experiment, respectively. Furthermore, transmission electron microscopy (Hitachi H-800, Hitachi, Tokyo, Japan) was used to determine the interior structure of NTs. A TEM sample was prepared by scratching the surface with a fresh razor blade and transferred onto a carbon coated copper supporting grid. The phase composition was checked using a room-temperature powder X-ray PANalytical X’Pert Pro MPD diffractometer (CuK_α_ λ 1.5406 Å). The surface composition was analyzed via X-ray photoelectron spectroscopy (XPS) using a PHI 5000 VersaProbeTM spectrometer (ULVAC-PHI, Chigasaki, Japan) with monochromatic Al Kα irradiation (hν = 1486.6 eV). The binding energy (BE) scale of all detected high-resolution (HR) spectra was referenced by setting the BE of the aliphatic carbon peak (C-C) signal to 284.8 eV. The photoluminescence (PL) measurements were taken at room temperature with an LS-50B Luminescence Spectrophotometer (Perkin Elmer LLC, Shelton, USA) with a Xenon discharge lamp as an excitation source and a special detector: an R928 photomultiplier. The sample’s surface was exposed to 360 nm of excitation radiation at a 90° angle. Using reference samples of barium sulphate, the diffuse reflectance spectroscopy (DRS) of the NTs was measured using a Shimadzu UV-Vis Spectrophotometer (UV 2600) (Shimadzu Corporation, Kyoto, Japan). Using a set scanning speed of 250 nm/min, spectra were measured in a range of 300–700 nm at room temperature. 

### 2.4. Measurement of Photocatalytic Bacteria Inactivation

The goal of the experiment was to investigate bacteria inactivation in the presence of photocatalysts. We performed (1) a photocatalysis test in the presence of a photocatalyst, light λ > 420 nm, and two control tests; (2) a dark process with the presence of a photocatalyst and a lack of light; and (3) a photolysis process that involved a lack of photocatalyst and the presence of light λ > 420 nm. A quartz reactor with a volume of 10 mL, a 1000 W Xenon Lamp (Oriel 66021, Oriel Instruments, Sterling, MA, USA), and a cutoff filter (>420 nm) were used to carry out the microorganism inactivation process. Irradiation intensity was measured in the position where light entered the photoreactor via an optical power meter (HAMAMATSU, C9536-01, Hamamatsu Photonics, Shizuoka, Japan) and equaled 100 mW/cm^2^. The following bacteria species were used in the experiment: *E. coli*, *S. aureus*, *K. oxytoca*, and *Clostridium* sp. Characteristics of the bacteria used in the experiment are included in Supplementary Materials (Table S1).

The dedicated protocol was followed to prepare all species of bacteria for the experiment. They were removed from LB medium via centrifugation at 2739× *g* for 10 min; then, they were suspended in sterile 1 × PBS (137 mM NaCl, 2.7 mM KCl, 10 mM Na_2_HPO_4_, 1.8 mM KH_2_PO_4_, pH 7.4, at 25 °C) with selected OD_600_ to achieve a final concentration of CFU/mL 10^3^–10^4^. The prepared photocatalytic layer was placed in the quartz reactor together with the bacteria suspension (8 mL). The reactor with a cooling system was located on a stirrer (500 rpm) and irradiated for 60 min. Starting with the reference collection, samples of 1 mL have been taken every 20 min. Serial dilutions in 1 × PBS from 10^−1^ to 10^−10^ were made from each sample. A total of 10 µL of the prepared dilutions was sown onto the agar medium, the plates were incubated for 16 h, and the grown colonies were counted. After incubation, grown colonies on agar plates were counted manually or using automatic colony counters. The number of bacterial colonies was then multiplied by 1/dilution and divided by the sample volume (in mL). CFU per unit volume (mL) of the original sample was calculated using the dilution factor and the number of colonies counted. The following formula to determine cultivated microorganisms was applied:

CFU/mL = number of colonies x total dilution factor/volume of culture plated in ml (CFU denotes the colony forming unit).

To perform the other control tests, the same setup was used. For the photolysis process (no catalyst control), only bacteria strains were placed in the reactor (without photocatalyst). Finally, for the experiment with no light control, the lamp was switched off. Additionally, during the bacteria inactivation processes, 1 mL samples of bacteria were taken for TEM imaging. Working on Tecnai G2 Spirit BioTWIN transmission electron microscope (Thermo Fisher Scientific, Waltham, MA, USA) with high resolution (HRTEM), the bacteria pictures were created to present the effects of photocatalytic processes.

To identify the irradiation range at which the most active sample maintained the capacity to inactivate microorganisms, a special setup was prepared where a sample with the photocatalyst and 8 mL of bacteria *S. aureus* were put in the quartz photoreactor with a cooling system and a magnetic stirrer bar. A total of 1 mL of solution was collected as a reference sample, and consecutive samples (1 mL) were taken after 2 h and 4 h. Using 1000 W Xe lamp (LSH602) and monochromator (MSW306) (LOT-Quantum Design, San Diego, CA, USA) the sample was irradiated with the following wavelengths: 420, 440, 460, 480, and 500 nm. Irradiation intensity (W) was measured for individual wavelengths with an optical meter (ILT2400, International Light Technologies, Peabody, MA, USA). 

The CO_2_ evolution during bacteria irradiation under visible light was studied to test the possibility of decomposition, i.e., mineralization. A photoreactor made of Teflon with a quartz window with a volume of 15 mL equipped with a cooling jacket, a 1000 W Xenon Lamp (Oriel 66021) (ArtisanTG, Champaign, IL, USA), and a cutoff filter (>420 nm) was used to carry out the microorganism inactivation process. The reactor’s temperature during experiments was maintained at 10 °C using a thermostat. The reactor was filled with 8 mL of 1 × PBS solution with *Staphylococcus aureus* at a concentration of CFU/mL = 4 ∗ 10^4^. Before the experiment, the headspace of the photoreactor was purged with nitrogen gas with 10 dm^3^/h velocity for 30 min in the dark while the solution was mixed with a magnetic stirrer bar. A total of 200 µL of the gas samples was taken after 0, 120, and 240 min of the process and then introduced into a gas chromatograph (Thermo Scientific TRACE 1300-GC, Waltham, MA, USA) that was equipped with a thermal conductivity detector (TCD). 

## 3. Results and Discussion

### 3.1. SEM and TEM Analysis

**Figure 1:**
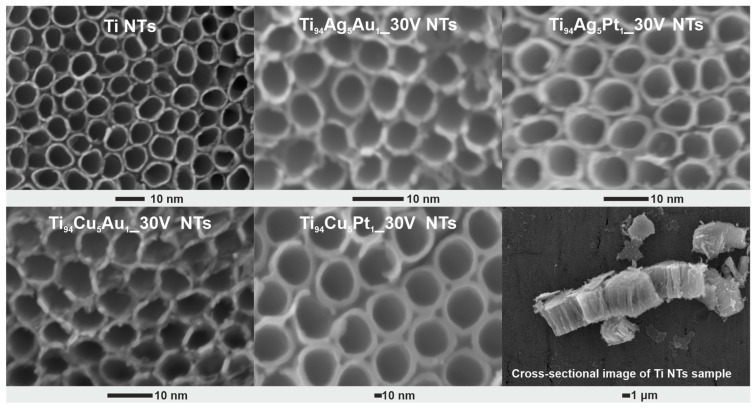
Top-view and selected cross-sectional HRSEM images of pristine and modified NT layers prepared from Ti and Ti alloys.

**Figure 2:**
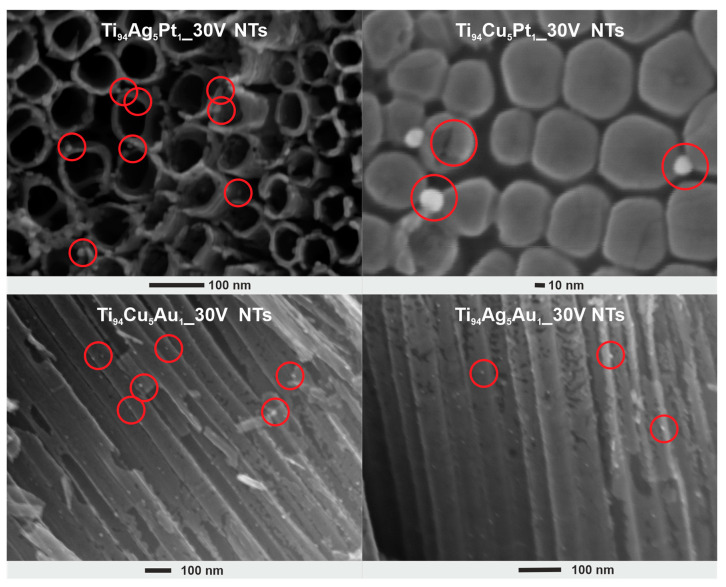
Selected HRSEM images showing the location of nanoparticles within the NT layers prepared from Ti alloys. The red circles indicate NPs.

Figure 1 presents HRSEM images of pristine and modified NT layers. It can be seen that regardless of the composition of the titanium alloy, the obtained NT layers are characterized by homogeneity, and their top surface is open (without the remains of the initiation layer). All NT layers consist of well-developed tubes placed side by side. The geometrical parameters of the obtained films determined based on SEM images are presented in Table 1. The NT layers obtained from titanium alloys were characterized by relatively similar thicknesses (length of NTs) and inner diameters ranging from 2.3 to 2.6 µm and from 54.3 to 58.8 nm, respectively. If we compare these parameters with the geometric dimensions of pristine NT layers, also given in Table 1, we can conclude that the small addition of other metals to titanium does not have a significant impact on the process of forming these nanotubular structures, and this is in line with the literature [24,29]. Furthermore, anodization of titanium substrates containing other metals such as Ag, Au, and Pt caused the formation of NPs, which are embedded within the entire surface of the NT layers; i.e., NPs are embedded on the upper surface, along the outer tubes, as well as inside and on their bottom, as shown in Figure 2.

**Figure 3:**
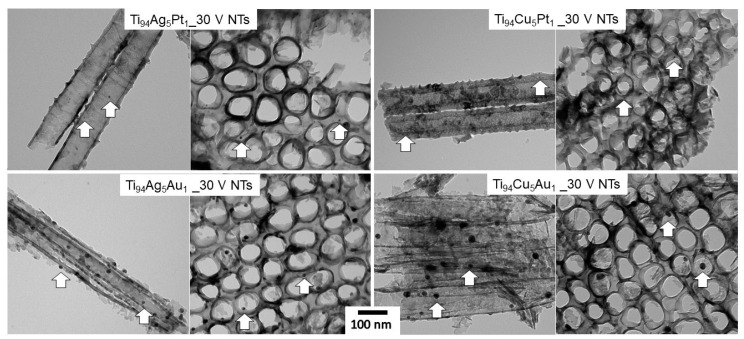
TEM images of modified NT layers prepared from Ti alloys. Exemplary nanoparticles of different sizes are marked by arrows.

TEM images (Figure 3 and Figure S1) also confirmed the presence of NPs in the layer of TiO_2_ NTs, where the nanoparticles (in the size range of 5–30 nm) had a spherical shape and were located on the surface and inside the NTs and were embedded in the NT walls. While in the layers obtained from Ti_94_Ag_5_Pt_1_ and Ti_94_Cu_5_Pt_1_ alloys, the nanoparticles show size of 8.2 ± 2.1 nm and 7.2 ± 1.5 nm, respectively, in the samples Ti_94_Ag_5_Au_1__30V NTs and Ti_94_Cu_5_Au_1__30V NTs, they show a much larger size, i.e., 14.1 ± 3.0 nm and 18.9 ± 6.2 nm, respectively.

### 3.2. X-ray Diffraction

**Figure 4:**
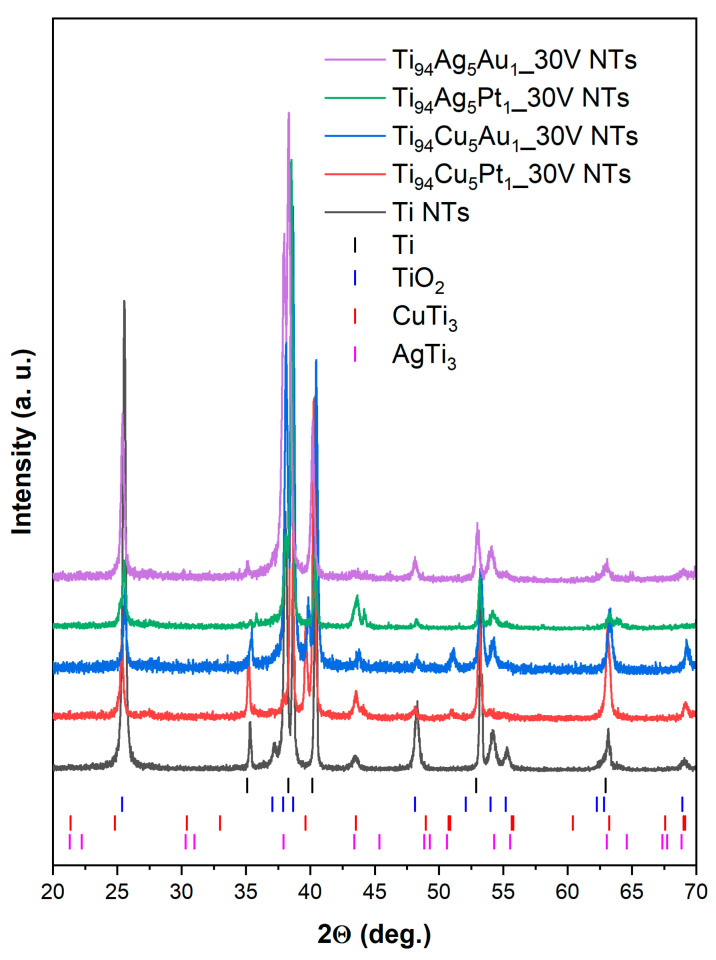
Powder X-ray diffraction (XRD) patterns of NT layers created using Ti and various Ti-alloy sheets. Vertical bars correspond to the Bragg positions of anatase phase (00-002-0387, ICDD)—blue; Ti (00-001-1198, ICDD)—black; AgTi_3_ (00-007-0249, ICDD)—pink; CuTi_3_—red (00-007-0109, ICDD).

Figure 4 displays the powder X-ray diffraction (XRD) patterns of NT layers created using Ti and various Ti-alloy sheets. The XRD patterns for both the pristine and modified samples revealed the existence of TiO_2_ anatase and Ti metal reflections, which were marked by the vertical bars: blue and black, respectively. The reflections at 2θ values of approximately 25, 38, 48, 53, and 54° corresponded to (101), (004), (200), (105), and (211) planes, which are particular to the anatase phase of TiO_2_ [35]. The reflections at 2θ values of around 35, 38, 40, and 54° originated from the hexagonal metallic Ti substrate [36]. Furthermore, the XRD patterns of NT layers created from different Ti-alloy sheets exhibited peaks of tetragonal AgTi_3_ and tetragonal CuTi_3_, as marked by the vertical bars: pink and red, respectively. These peaks, like the reflections of titanium, originated from the Ti-alloy substrates [26].

### 3.3. Raman Spectroscopy 

**Figure 5:**
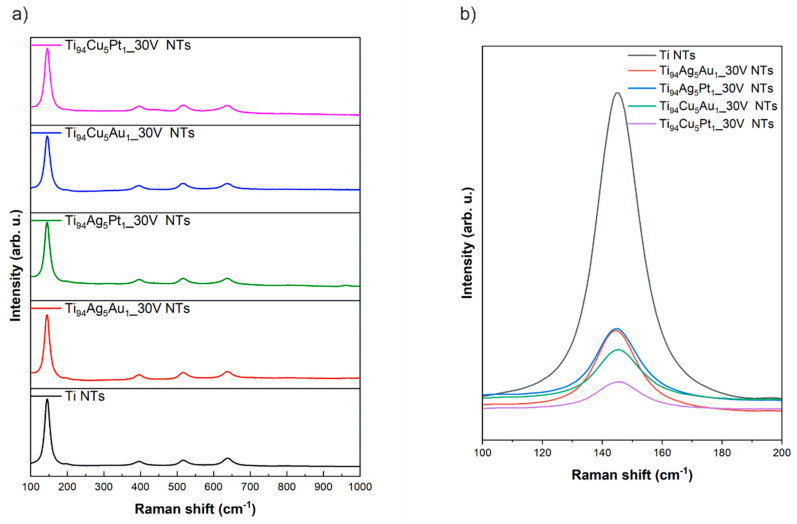
General Raman spectra of pristine and modified NT layers (**a**) and Raman spectra showing Eg_(1)_ active modes (**b**).

Figure 5a shows Raman spectra of pristine and modified NT layers prepared from Ti and Ti alloys. Raman spectra of pristine and modified NT layers contained five signals assigned directly to the anatase crystalline [37] form, which were located at 143, 196, 391, 514, and 634 cm^−1^ and are derived from Eg_(1)_, Eg_(2)_, B_1g_, A_1g_, and Eg_(3)_ active modes, respectively [38]. The Raman spectra recorded for both types of NTs layers (pristine and modified) were almost the same. No signal from Ag or Cu or Pt and Au or their oxide forms was recorded in the spectrum, which may be due to their very small quantity in the nanotube layer. However, the location of the Eg_(1)_ for the NT layers obtained from titanium alloys differs in comparison with the NT layer prepared from pure Ti substrate, as shown in Figure 5b. The Eg_(1)_ mode is typically ascribed to the symmetric stretching vibration of O–Ti–O in TiO_2_ materials [39]. In modified NT layers, a slight blue shift in the position of the Eg_(1)_ mode was observed in comparison with pristine NTs. This could be related to the non-stoichiometry inducing lattice vibrations. The appearance of oxygen vacancies, resulting from introducing modifiers, can also cause the Ti–O–Ti disturbances observable as a displacement of the Eg_(1)_ peak towards higher wavenumbers [40].

### 3.4. DRS and Photoluminescence 

**Figure 6:**
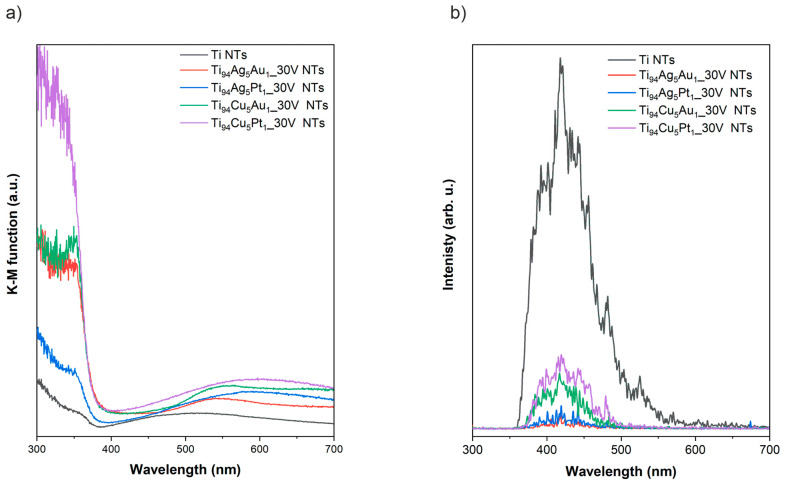
(**a**) DRS and (**b**) photoluminescence spectra of pristine and modified NT layers.

Diffuse reflectance spectroscopy in the UV-Vis range for all prepared NT layers is presented in Figure 6a. The strongest photoabsorption of light, regardless of the type of substrate used during anodizing, was observed for the range of UV irradiation up to 380 nm typical for TiO_2_ NTs in the crystallite form of anatase, which is caused by the electronic transition between bands in the anatase structure [41]. Additionally, in the cause of pristine NT layers, the photoabsorption band in the visible range with a maximum at approximately 500 nm could be attributed to the presence of sub-bandgap states as a result of nanotubular morphology [42]. The shapes of the photoabsorption curves in the visible range recorded for NTs layers obtained from titanium alloys differ significantly compared to the pristine NT layers. The photoabsorption in the visible light of modified NT layers is mainly caused by the narrowband oxide form of Ag and Cu [13,43]. Furthermore, an absorption band with a maximum of approximately 540 nm was observed in the case of NT layers containing Au NPs, which could be related to the presence of Au NTs within NT layers [44]. 

The photoluminescence spectra recorded in the range of 300–700 nm for all prepared NT layers is presented in Figure 6b. Emissions with the maximum at approximately 420, 445, 480, and 530 nm can be assigned to the TiO68-, surface defects, oxygen vacancies, and radiative recombination [45,46]. In general, lower PL intensity is ascribed to the lower rate of charge carriers recombination [47]. In this respect, NT layers obtained on titanium alloys were characterized by slower charge carriers recombination process compared to pristine NTs. On the other hand, among the modified samples, this process was the most effective in samples prepared from Ti_94_Ag_5_Pt_1_ and Ti_94_Ag_5_Au_1_ substrates. 

### 3.5. X-ray Photoelectron Spectroscopy (XPS)

**Figure 7:**
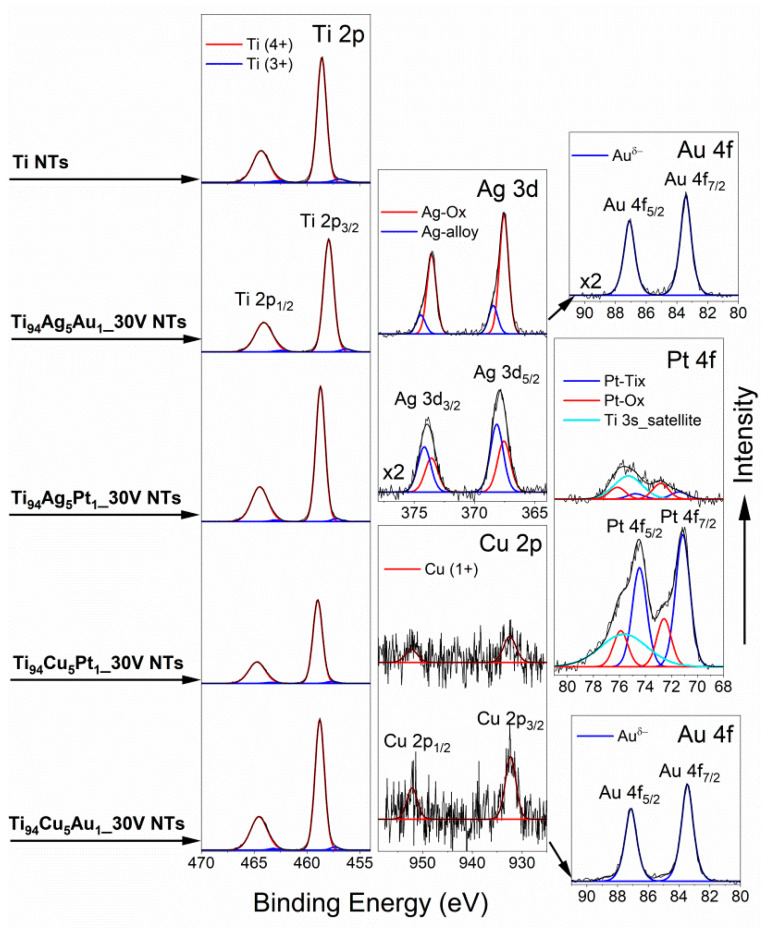
High-resolution Ti2p, Ag3d, Cu 2p, Pt 4f, and Au 4f XPS spectra identified on Ti NTs and various NT layers prepared from Ti-alloy sheets.

The surface chemical composition of anodized Ti- and Ti-alloy sheets was identified from survey XPS spectra (Figure S2) and determined after analysis of high-resolution (HR) XPS spectra, and the results are summarized in Table 2. The HR XPS spectra of metal alloys components, presented in Figure 7, confirm the presence of oxidized states of Ti, Ag, Cu, and Pt. Two chemical states of titanium (identified as Ti^4+^ and Ti^3+^), identified in the Ti 2p spectra (BE of Ti 2p_3/2_ peak close to 458.8 eV and 457.3 eV, respectively) confirm the effective formation of TiO_2_] [48,49]. The Ag 3d_5/2_ signals at 367.6 eV and 368.5 eV in the Ag 3d spectra of the Ti_94_Ag_5_Au_1__30V NTs Ti_94_Ag_5_Pt_1__30V samples are related to Ag-Ox and Ag-alloy, respectively [48]. The Cu 2p spectra of Ti_94_Cu_5_Pt_1__30V NTs and Ti_94_Cu_5_Au_1__30V NTs samples reveal the Cu^1+^ state of copper oxide (Cu 2p_3/2_ signals at 932.4 eV [48,49]). The Pt 4f spectra of the Ti_94_Cu_5_Pt_1__30V NTs and Ti_94_Ag_5_Pt_1__30V NTs samples are overlapping with the broad Ti 3s satellite. However, the Pt states, shown by the Pt 4f_7/2_ signals at BE 71.5 and 73.0 eV, can be addressed to the Pt-Tix [48,50] and Pt-Ox surface species [48]. The Au 4f spectra of Ti_94_Ag_5_Au_1__30V NTs and Ti_94_Cu_5_Au_1__30V NTs samples (Au4f_7/2_ and Au4f_5/2_ signals at 83.4 eV and 87.1 eV, respectively) are related to the electronegative (Au^δ−^) state [51,52]. The O/Ti atomic ratios for all Ti-alloy samples are close to or slightly higher than the corresponding values for Ti NTs (Table 2). However, the relative proportions of metals in the surface region of NTs are significantly different from the corresponding values of starting alloy materials (Table 3). This is a result of different surface segregations of Ti, Ag, Cu, and Au atoms during the anodic treatment of Ti-alloy samples, resulting in different surface reactivities with oxygen and CO. The data in Table 2 show that the Au content on the surface of the most catalytically active Ti_94_Ag_5_Au_1__30V NTs sample is higher than that of the initial alloy sample (see Ti/Au and Ag/Au ratios), indicating the surface segregation of Au both in relation to Ti and Ag during the anodization process.

### 3.6. Photocatalytic Antibacterial Activity

#### 3.6.1. Effectiveness of Bacterial Inactivation

The experiment was performed to check the efficiency of bacterial inactivation in the presence of the obtained photocatalyst. To prove this, three types of experiments were defined. First, we checked whether the photocatalyst had an effect on bacteria without light application. We assumed that some metal ions can have an impact on the bacteria’s cell walls. This analysis showed that nanotubes are not harmful to bacteria when they are not exposed to irradiation. Only in the case of two samples, Ti_94_Ag_5_Au_1_ NTs in *K. oxytoca* solution and Ti_94_Cu_5_Au_1_ NTs in *E. coli* and *Clostridium* sp., did the number of bacteria slightly decrease. This effect could be caused by the influence of silver or copper oxides located on the surface of nanotubes made of TiO_2_/Ag_2_O/Au^0^ and TiO_2_/Cu_2_O/Au^0^. In previous experiments in the presence of TiO_2_/Cu_2_O/CuO nanotubes, we checked whether and how many Cu ions are transferred directly from the sample to the bacterial solution, and the result confirmed the absence of such a process [12].

To confirm that photolysis does not influence the microorganisms, bacterial solutions without photocatalyst were examined with visible range irradiation (λ > 420 nm) for 60 min. It was proven that there is no impact of light on *E. coli*, *S. aureus*, *K. oxytoca*, and *Clostridium* sp. in these conditions.

**Figure 8:**
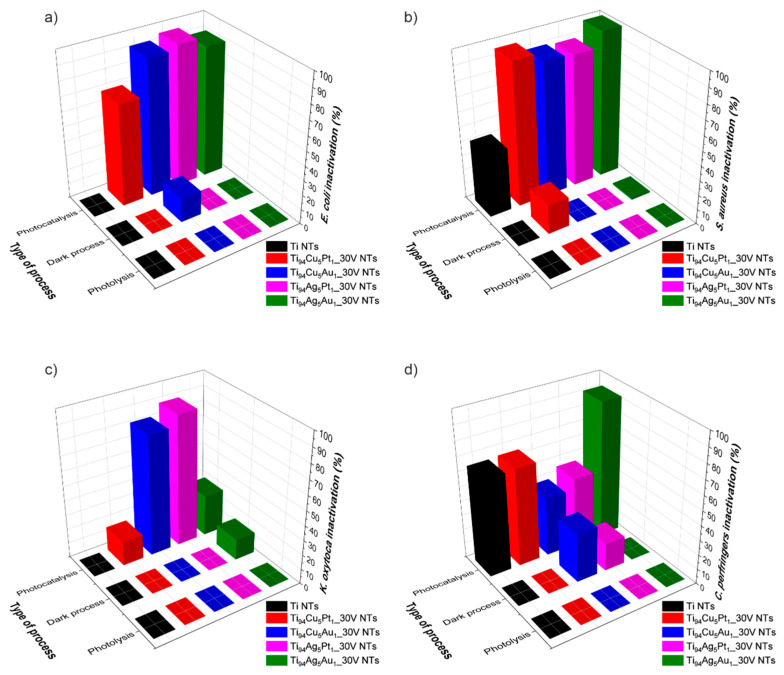
Effectiveness of inactivation for bacteria: (**a**) *E. coli*, (**b**) *S. aureus*, (**c**) *K. oxytoca*, and (**d**) *Clostridium* sp. Type of the process: photocatalysis—presence of photocatalyst and light λ > 420 nm; dark process—presence of photocatalyst, lack of light; photolysis—lack of photocatalyst, presence of light λ > 420 nm.

The focus of the final series of studies was on assessing how visible-light-driven photocatalysis affected the survival of bacteria in the 60 min cycle. The effectiveness of inactivation for all bacteria, showing the influence of the type of formed heterostructure, is presented in Figure 8. The most promising results were obtained for samples Ti_94_Ag_5_Au_1_ NTs and Ti_94_Ag_5_Pt_1_ NTs. When bacteria were exposed to irradiation over Ti_94_Ag_5_Au_1_ NTs, *E. coli* and *S. aureus* bacteria were the most effectively destroyed (90% and 99.9%, respectively), and *Clostridium* sp. demonstrated a modest drop (92.8%). In the case of Ti_94_Ag_5_Pt_1_ NTs, the results were slightly lower; *E. coli* and *S. aureus* and *K. oxytoca* were 96.5%, 90%, and 82.5% destroyed, respectively. When analyzing each bacterium, *E. coli* was degraded in the visible light range in the presence of Ti_94_Ag_5_Pt_1_ NTs by 96.5% efficiency, Ti_94_Cu_5_Au_1_ NTs by 93.3%, and Ti_94_Ag_5_Au_1_ NTs by 90%. Experiments for *S. aureus* show a level of degradation for Ti_94_Ag_5_Au_1_ NTs of 99.9%, somewhat less for Ti_94_Cu_5_Pt_1_ NTs (i.e., 97%), and 91% for Ti_94_Cu_5_Au_1_ NTs. The analysis conducted for *Clostridium* sp. presented a high influence of Ti_94_Ag_5_Au_1_ NTs on bacteria (92.8% degradation), 66.7% for Ti_94_Cu_5_Pt_1_ NT, and a lower impact of Ti_94_Ag_5_Pt_1_ NTs (47%) and Ti_94_Cu_5_Au_1_ NTs (40%). The bacterium that is the least affected by the photocatalysis process and the tested materials is *K. oxytoca*. The highest degradation rate was 82.5% for Ti_94_Ag_5_Pt_1_ NTs; however, for other materials, it was less than 35%. 

When we analyze the composition of the most active photocatalysts, Ti_94_Ag_5_Au_1__30V NTs and Ti_94_Ag_5_Pt_1__30V NTs, it is known that both samples contain silver. XPS analysis confirmed the existence of Ag phases in amounts of 0.47 and 0.23 atomic %, and these different amounts of Ag_2_O can affect the charge transfer and recombination processes, which are crucial for photocatalytic activity. Moreover, the surface plasmon resonance effect of Au NPs can enhance the absorption of visible light, which is not efficiently absorbed by the semiconductor alone, thus increasing the photocatalytic activity. Ti_94_Ag_5_Au_1__30V NTs and Ti_94_Ag_5_Pt_1__30V NTs also show significantly reduced photoluminescence (of similar value) compared to pristine TiO_2_ NTs, as well as the remaining samples. The reduced value of the photoluminescence maxima suggests that the electron–hole recombination process is slower, which might increase the photocatalytic properties of these materials as well. XPS analysis also shows that there is more atomic % of Ag than Cu, which can lead to more effective bacterial inactivation for certain bacteria. Silver ions are more potent than copper ions at lower concentrations, which may contribute to the higher inactivation rates observed with higher atomic percentages of Ag [53].

The different activity observed per bacterium can be the result of differences in cell wall structure between Gram-positive (*Staphylococcus aureus*, *Clostridium* sp.) and Gram-negative bacteria (*Escherichia coli*, *Klebsiella oxytoca*), which could highly affect their susceptibility to photolysis. Gram-negative bacteria typically have an outer membrane that may provide additional protection against photolysis compared to Gram-positive bacteria. However, some Gram-positive bacteria generate endospores, which are resistant to external environmental factors. Moreover, the bacterium *Klebsiella oxytoca* can create an additional protective layer, a polysaccharide coating that prevents degradation. Variations in metabolic pathways and the ability to regenerate cellular components among bacterial species may also affect their susceptibility to photolysis. Bacteria with more robust antioxidant systems or DNA repair mechanisms are expected to be less susceptible to photolysis-induced damage. Certain bacteria may produce extracellular polymeric substances (EPS) (*Klebsiella oxytoca*) or biofilms that could shield them from the effects of photolysis. Additionally, certain bacterial species may have mechanisms to detoxify reactive oxygen species (ROS) generated during photolysis. Variations in bacterial genetics, including the presence and expression levels of specific genes involved in stress response or DNA repair pathways, could also contribute to differences in sensitivity to photolysis.

During the study of bacterial degradation under visible light, the Ti_94_Ag_5_Au_1_ NTs sample was used an average of 25 times for both processes involving photocatalysts ((1) photocatalysis test in the presence of a photocatalyst and light λ > 420 nm; (2) a dark process in the presence of a photocatalyst and a lack of light). The results of the photocatalysis processes performed in the presence of the Ti_94_Ag_5_Au_1_ NTs sample in eight reuse cycles are gathered in Figure S3. There was no drop in photoactivity; therefore, it can be assumed that these materials are stable over time.

Many studies have been described in the literature regarding the inactivation of *E. coli* using visible light in the presence of various types of photocatalysts, including Au/TiO_2_ [54], Ag/TiO_2_ [55], Cu/TiO_2_ [56], AgVO_3_/g-C_3_N_4_, BiVO_4_ [57], and many more. On the contrary, studies on *S. aureus* elimination have not been conducted so extensively. This is likely due to the characteristics of this bacterium, as certain strains exhibit antibiotic resistance, making their elimination more challenging. Table 4 presents examples of photocatalysts that demonstrate high bacterial killing efficiency during the photocatalysis process. Among others, Moon et al. [58] reported the development of visible light antibacterial TiO_2_ nanotubes modified with Au and Pt NPs. Their study demonstrated that Au-modified TiO_2_ nanotubes exhibited a higher inactivation efficiency, achieving a rate of <50% at an initial bacterial concentration of 10^5^ CFU/mL. On the other hand, the data for modification of NT with Pt NPs revealed an inactivation efficiency of <40%. A composite material in the form of MoS_2_/TiO_2_ nanotubes was utilized in a photocatalytic process for the inactivation of *S. aureus* [18]. The process was carried out for a duration of 150 min, leading to a remarkable inactivation efficiency of 100%. Interestingly, a sol–gel method was employed to synthesize a visible-light-active material consisting of Cu-doped TiO_2_ [59]. It was found that the addition of a small amount of Cu into the TiO_2_ crystal lattice enhanced thermal stability and antimicrobial properties. Cu-TiO_2_ achieved complete inactivation of *S. aureus* within just 30 min of visible light exposure. In conclusion, the existing literature, aside from the research conducted by the authors, lacks examples of the utilization of ternary photocatalysts in the form of NTs for the elimination of *S. aureus*. Furthermore, while many of the materials highlighted in Table 4 demonstrate high process yields, their sample preparation and process duration are significantly more complex compared to the materials presented in the present study. The photocatalysts analyzed in this research were prepared using a single-stage anodization process, enabling easy acquisition of visible-light-active structures while also offering reusability for multiple cycles.

#### 3.6.2. Mechanism of Bacterial Inactivation

**Figure 9:**
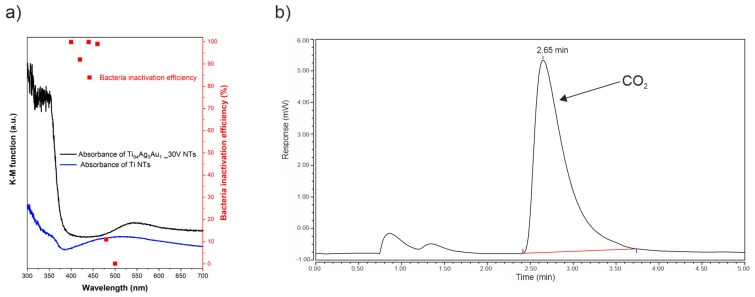
(**a**) Effectiveness of inactivation of *S. aureus* during the photocatalytic reaction in the presence of Ti_94_Ag_5_Au_1__30V alloy at variable wavelengths (monochromatic light source); (**b**) chromatogram showing CO_2_ generation after 4 h visible light irradiation of *S. aureus* in the presence of Ti_94_Ag_5_Au_1__30V sample.

To determine the range of irradiation in which the most active sample still showed the ability to inactivate bacteria, we conducted experiments wherein *S. aureus* bacteria, in the presence of the Ti_94_Ag_5_Au_1__30V NTs sample, were irradiated with wavelengths of 420, 440, 460, 480, and 500 nm. The results are presented in Figure 9a. The measurements demonstrated that Ti_94_Ag_5_Au_1__30V NTs are characterized by strong photoactivity in the visible range up to 460 nm, with a value of 99.4% after 4 h which diminished, reaching a value of 0% at 500 nm. This phenomenon can be explained by the insufficient power of irradiation above 500 nm to effectively induce the generation of charge carriers in Ag_2_O. Essentially, irradiation nearer to the UV spectrum possesses higher energy, which seems to be crucial for the effective excitation of Ag_2_O and subsequent generation of charge carriers. Additionally, to explain the mechanism of the photocatalytic inactivation of bacteria in the field of visible irradiation, measurements of CO_2_ generated during the bacterial inactivation process were also carried out. An increase in the amount of CO_2_ generated was observed during the 4 h measurement (Figure 9b, Table S2), which can prove that carbon dioxide production by *S. aureus* bacteria may be a result of bacterial mineralization or metabolic and respiratory processes [69,70].

**Figure 10:**
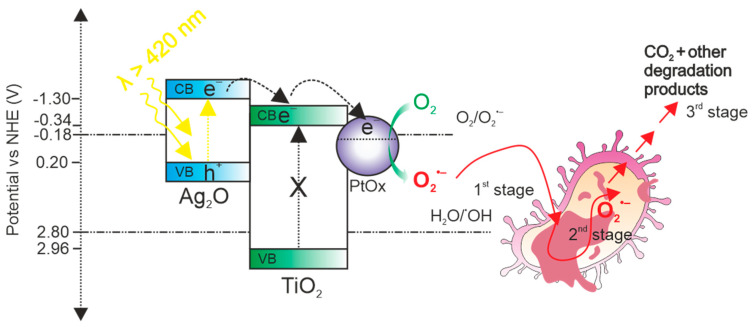
The simplified excitation mechanism of TiO_2_/Ag_2_O/PtO_x_ NTs under visible light and the proposed stages of photocatalytic bacteria inactivation.

First, the excitation mechanism should be mentioned to understand, comprehensively, what happens to bacteria during photocatalytic experiments where radical oxygen species (ROS) are formed. Based on literature data, Figure 10 reveals a schematic excitation mechanism under visible irradiation, where one of the two most active photocatalysts in bacterial inactivation, TiO_2_/Ag_2_O/PtO_x_, was examined.

It is known that TiO_2_ alone cannot be excited in the visible light (λ > 420 nm). The reason for this is its wide gap energy, with the value of 3.2 eV for anatase, which correlates with wavelength of 387 nm. The irradiation from the visible range is not able to excite electrons from the valence band of TiO_2_ as the radiation energy is smaller than the energy bandgap. However, the anodization of the Ti-Ag alloy led to the formation of not only TiO_2_ but also Ag_2_O (with a bandgap as narrow as 1.3 eV [13]) [35], which, due to the narrow energy bandgap, can be excited by visible light. Moreover, it is confirmed that plasmon resonance is responsible for the visible activity of wide-bandgap semiconductors [71,72]. Ag_2_O from the synthesized heterojunction becomes excited while absorbing visible light energy and generates a pair of free electrons (e−) in the conduction band (CB) and holes (h+) in the valance band (VB) that can migrate to the surface of the photocatalyst, where reactions take place. As shown in Figure 10, the electrons move from the Ag_2_O to the TiO_2_ CB because the CB of Ag_2_O has a greater negative potential than the CB of TiO_2_ [73]; then, electrons transfer to the PtO_x_, causing better electron–hole separation [24,74,75]. The free electrons whose reduction potential is sufficient (more negative −0.18 eV) react with oxygen molecules to form superoxide ions (O_2_^•−^). The potential of holes in the VB of Ag_2_O is too low (0.20 eV) to generate strong oxidants such as ^•^OH; thus, it is proposed that these holes can directly participate in the process of bacteria inactivation [76]. However, it is known that the efficiency of oxidation reactions in photocatalysis is influenced by the oxidative potential of the holes [77]. In the case of Ag_2_O, the oxidative potential of the holes is low (0.20 eV); therefore, they can only have a minor effect on bacterial inactivation. Several scenarios can be true in terms of what happens to bacteria when photocatalysis is involved. In the course of photocatalytic reactions, bacteria cells can be rendered inactive by a variety of mechanisms, including membrane disruption (rupture of the cell membrane), cell wall exposure (exposure of cellular components), and ROS attack on the cells [78]. It should be noted that photocatalyst intrinsic and surface features (e.g., bandgap energy, specific surface area, crystallite/particle size, aggregation, surface charge, defects distribution, and impurities content) are equally important for the overall antimicrobial action. The antimicrobial activity of nanoparticles modified by noble metals has been reported in many studies, presenting several pathways of bacteria damage. For structures involving silver NPs, suggested mechanisms include adsorption of Ag cations on a negatively charged bacterial cell wall [79], interaction with enzymes’ thiol groups [80], and the possibility of membrane breakdown [81]. Because of its strong photocatalytic activity (ROS production) [82] and capacity to impair electron transport in cells [83], the mechanism involving Au-modified titania has also been studied. For cooper NTs, the presumed antimicrobial mechanisms involve copper ion accumulation inside bacteria [84], the denaturation of surface protein structure [85], but also the generation of oxidative stress in bactericidal processes by absorbed copper ions [86]. All of the mentioned reports on antimicrobial activity can be helpful in discussing the possible mechanism; however, they provide information only for binary complexes. The presented results indicate that the mechanism of bacterial inactivation consists of two steps. The first step, the shorter one, covered the bacterial cell wall, and in Gram-negative bacteria, the outer cell membrane was also covered, and the inactivation took place as a result of the action of oxygen radicals (O_2_^•−^) produced during the photocatalytic process. The low amount of CO_2_ indicates that only some of the bacteria’s protective elements have been compromised. It can be assumed that at this stage, the bacteria try to repair themselves and create a mechanism by which to defend themselves against radicals [87]. Since only later in the process (after approx. 40 min) was a significant increase in the efficiency of bacterial inactivation found, it can be concluded that at this point, the bacteria stopped defending themselves, and the second stage, mineralization, began in the inactivation mechanism. The release of carbon dioxide may also indicate bacteria metabolic and respiratory processes. Radicals attacked the cell wall and membrane and slowly caused the oxidation of more complex compounds such as liposaccharides (LPS) [88].

In both cases, the high-performance material contained silver in various forms. Therefore, it has been assumed that the membranes of both Gram-positive and Gram-negative bacteria are exposed to Ag NPs because of the surface oxidation process. Electrostatic interaction between the composite particles and the bacterial cell wall may also cause inactivation. What is more, the diameter of the metallic nanoparticles can influence the process because smaller nanoparticles with a greater surface area during their interaction with bacterial cells lead to the better penetration of cytoplasm compared to larger particles [89]. The slightly lower effectiveness for *E. coli* (Gram-negative bacteria) could result from the structure of the microorganisms’ membranes as they are built from phospholipid and lipopolysaccharide layers, which makes them harder to penetrate.

**Figure 11:**
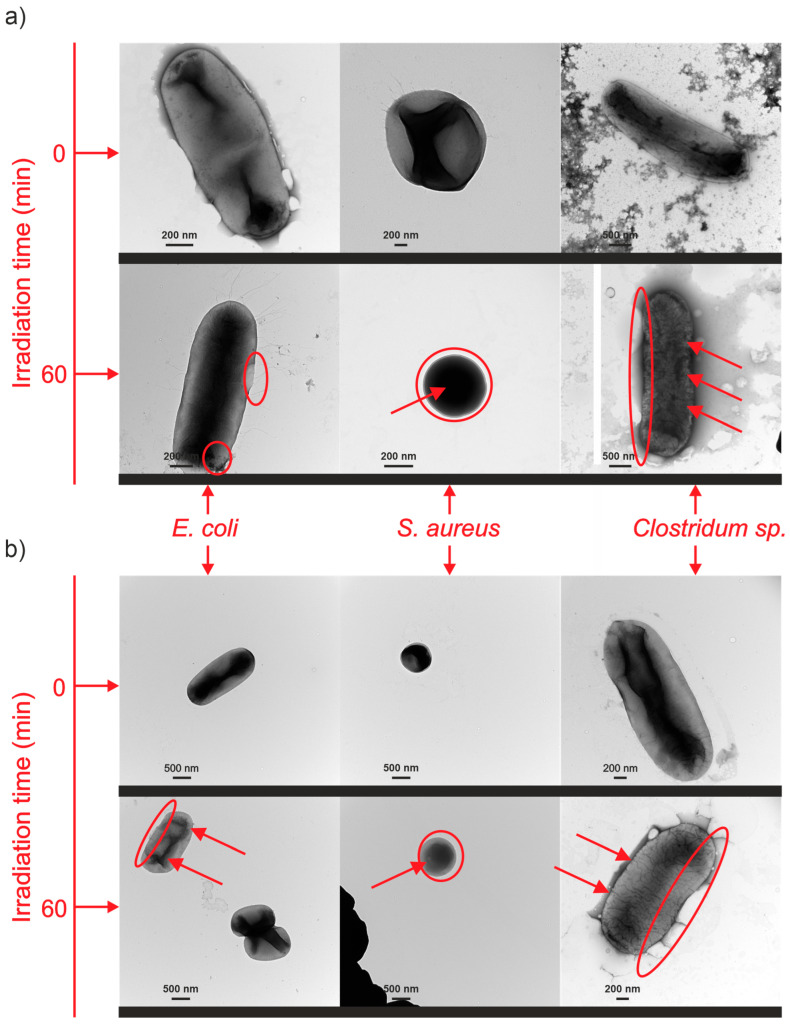
TEM images of bacteria after 0 and 60 min of visible light irradiation with (**a**) Ti_94_Ag_5_Au_1__30V and (**b**) Ti_94_Ag_5_Pt_1__30V NTs photocatalysts. The red circles indicate the disintegration of the cell wall/membrane, i.e., the visible cracks and damage to the bacteria around the cell. The red arrows mark the destruction of the cell interior, i.e., the destruction of the structure of internal organelles, and the visible scattered or damaged cytoplasmic structures or fragmentation of genetic material.

For selected alloys, TEM images of bacteria were taken after 60 min of the photocatalytic process. Figure 11 shows pictures of bacteria in the presence of the most active nanomaterials: Ti_94_Ag_5_Au_1__30V NTs and Ti_94_Ag_5_Pt1_30V NTs. The images clearly show damage to the bacteria’s cell walls and nucleus.

## 4. Conclusions

We propose the fabrication of highly visible-light-active nanotube layers composed of TiO_2_/Ag_2_O/PtO_x_, TiO_2_/Ag_2_O/Au^0^, TiO_2_/Cu_2_O/Au^0^, or TiO_2_/Cu_2_O/PtO_x_ by utilizing a simple one-step anodization method on Ti alloys. The resulting nanotube (NT) layers were characterized by similar lengths (ranging from 2.3 to 3.0 nm) and shapes, with nanoparticles (NPs) distributed on the surface and within the walls of the nanotubes. Inactivation of bacteria in the presence of NT layers obtained from alloys under visible light irradiation has been confirmed via the following methods: (i) counting bacterial colonies; (ii) observing the bacteria under a microscope; and (iii) confirming that CO_2_ is released. All NT layers presented antibacterial activity under visible light radiation, but the most active materials were TiO_2_/Ag_2_O/Au^0^ NTs, with an efficiency of 99.9% in the presence of *S. aureus*, TiO_2_/Ag_2_O/PtO_x_ NTs, with 96.5% effectiveness on *E. coli*. The observed significant antibacterial activity of the NT layers results from the combined effects of two phenomena: the direct interaction of silver/copper species and the photocatalytic reaction. Additionally, the mechanism of bacteria inactivation was studied, which revealed that oxygen radicals (•O_2_^−^) react with the walls and membranes of microorganisms, causing their cracking and, consequently, their inactivation. Photocatalytic inactivation of *E. coli*, *S. aureus*, and *Clostridium* sp. was confirmed by TEM imaging of bacterium cell destruction and the detection of CO_2_ as a result of bacterial cell mineralization or metabolic and respiratory processes for the most active sample. These results suggest that the membrane ruptures as a result of the attack of active oxygen species, and then, both the membrane and the contents are mineralized to CO_2_.

The obtained materials are promising for further research and application in various branches of industry as they are easy to use multiple times in photocatalytic processes and it is possible to scale them up in a simple way. Moreover, the obtained photocatalysts confirmed the removal of types of bacteria that are dangerous for humans, which is very promising for further studies on the antimicrobial activity of photocatalysts.

## Figures and Tables

**Table 1:** Sample label, main anodization conditions, and dimensions of nanotubes prepared at different Ti-alloy sheets.

Sample Label	Composition of Alloy (wt. %)	Synthesis Parameters		Morphology of Obtained Nanotubes Based on SEM Imaging	
		Voltage (V)	Time (min)	Inner diameter (nm)	Length (µm)
Ti NTs	100% Ti	30 V	90 min	64.3 ± 4.9	2.6 ± 0.1
Ti_94_Ag_5_Au_1__30V NTs	94% Ti, 5% Ag, 1% Au	30 V	60 min	58.8 ± 6.8	2.3 ± 0.1
Ti_94_Ag_5_Pt_1__30V NTs	94% Ti, 5% Ag, 1% Pt	30 V	60 min	54.3 ± 5.5	2.5 ± 0.2
Ti_94_Cu_5_Au_1__30V NTs	94% Ti, 5% Cu, 1% Au	30 V	60 min	55.2 ± 6.2	2.6 ± 0.1
Ti_94_Cu_5_Pt_1__30V NTs	94% Ti, 5% Cu, 1% Pt	30 V	60 min	57.5 ± 6.2	2.5 ± 0.1

**Table 2:** Elemental composition (in atomic %) in the surface layer of Ti NTs, Ti_94_Ag_5_Au_1__30V NTs, Ti_94_Cu_5_Pt_1__30V NTs, Ti_94_Cu_5_Au_1__30V NTs, and Ti_94_Ag_5_Pt_1__30V NTs composites evaluated via XPS analysis.

Sample Label	Elemental Composition (Atomic %.)								
	Ti	O	C	N	Cu	Ag	Pt	Au	O/Ti
Ti NTs	24.23	62.16	12.70	0.91	-	-	-	-	2.56
Ti_94_Ag_5_Au_1__30V NTs	24.40	63.19	10.95	0.62	-	0.47	-	0.38	2.59
Ti_94_Cu_5_Pt_1__30V NTs	22.12	62.22	13.65	1.41	0.11	-	0.49	-	2.81
Ti_94_Cu_5_Au_1__30V NTs	24.56	62.56	11.45	0.59	0.16	-	-	0.68	2.55
Ti_94_Ag_5_Pt_1__30V NTs	24.07	64.35	10.56	0.74	-	0.23	0.05	-	2.67

**Table 3:** Surface metal composition of Ti NTs and different NT layers prepared from Ti-alloy sheets evaluated via XPS analysis.

Sample Label	Ti/Me Ratio Based on XPS Analysis (Nominal Ti/Me Ratio of Ti-Alloys)				Me_5_/Me_1_ Ratio Based on XPS Analysis (Nominal Me_5_/Me_1_ Ratio of Ti-Alloys)				Ti 2p_3/2_ Fraction (%)	
	Ti/Ag	Ti/Cu	Ti/Pt	Ti/Au	Ag/Au	Cu/Pt	Cu/Au	Ag/Pt	Ti(^4+^) 458.8 ± 0.2 eV	Ti(^3+^) 457.3 ± 0.3 eV
Ti_94_Ag_5_Au_1__30V NTs	52.1 (18.8)	-	-	64.2 (94)	1.2 (5)	-	-	-	97.30	2.70
Ti_94_Cu_5_Pt_1__30V NTs	-	201.1 (18.8)	45.1 (94)	-	-	0.2 (5)	-	-	97.36	2.64
Ti_94_Cu_5_Au_1__30V NTs	-	153.5 (18.8)	-	36.1 (94)	-	-	0.2 (5)	-	97.70	2.30
Ti_94_Ag_5_Pt_1__30V NTs	104.7 (18.8)	-	481.4 (94)	-	-	-	-	4.6 (5)	97.76	2.24
Ti NTs	N/A				N/A				96.57	3.43

**Table 4:** Photocatalysts used for *S. aureus* inactivation under visible light.

Photocatalyst Type	Form	Preparation Method	Time of the Irradiation (min)	The Initial Concentration of Bacteria (CFC/mL or UFC/mL)	Inactivation Efficiency (%)	Ref.
Ag/TiO_2_	nanoparticles	Template induction	-	10^6^	100	[60]
Cu/TiO_2_	particles	Sol–gel	-	-	99.9	[59]
Au/TiO_2_	nanotubes	Magnetron sputtering	-	10^5^	<50	[58]
Pt–TiO_2_	nanotubes	Photodeposition	-	10^5^	<40	[58]
F-ZnO	particles	Sol–gel	360	-	99.9	[61]
Ti- BiOI	particles	Solvothermal method	45	3 × 10^6^	100	[62]
AgI@MnO_2_	particles	Deposition	25		99.4	[63]
MoS_2_/TiO_2_	nanotubes	Two-step anodization and hydrothermal method	150	>10^8^	100	[18]
g-C_3_N_4_-V-TiO_2_	particles	Hydrothermal calcination	60	-	99.5	[64]
ZnCl_2_/TiO_2_	nanoparticles	Sol–gel calcination	120	10^5^–10^6^	>90	[65]
Zn(Ac)_2_/TiO_2_	nanoparticles	Sol–gel calcination	120	10^5^–10^6^	>80	[65]
Zn(NO_3_)_2_/TiO_2_	nanoparticles	Sol–gel calcination	120	10^5^–10^6^	>95	[65]
ZnSO_4_/TiO_2_	nanoparticles	Sol–gel calcination	120	10^5^–10^6^	100	[65]
TiO_2_–SiO_2_	nanocomposite	Sonochemistry method	120	-	98.6	[66]
Cu–TiO_2_/GF	particles	Immersion–drying process	-	-	67.49	[67]
g-C_3_N_4_/Cu@CdS	nanocomposite	Co-precipitation method	-	-	40	[68]
TiO_2_/Ag_2_O/Au	nanotubes	One-step anodization	60	10^3^–10^4^	99.9	This work

## Data Availability

The data presented in this study are available on request from the corresponding author.

## Supplementary Materials

The following supporting information can be downloaded at: https://www.mdpi.com/article/10.3390/nano14050409/s1, Figure S1. TEM images of modified NT layers prepared from Ti alloys; Figure S2. Survey XPS spectra identified on Ti NTs and various Ti-alloy sheets; Figure S3. Photocatalysis processes performed in the presence of Ti94Ag5Au1 NTs sample in 8 reuse cycles; Table S1. Characteristics of bacteria used in the experiments; Table S2. The concentration of CO_2_ (μmol/L) varied in the reactor’s headspace during the photocatalytic inactivation of bacteria, depending on the specific process utilized. Refs. [90,91,92,93,94,95] are cited in Supplementary Materials.
