# Supplementary material for: Detailed Insight into Photocatalytic Inactivation of Pathogenic Bacteria in the Presence of Visible-Light-Active Multicomponent Photocatalysts
Source: Nanomaterials (Basel). 2024 Feb 23;14(5):409. doi: 10.3390/nano14050409 (PMC10934703; doi:10.3390/nano14050409)
Supplement: Supplementary file 1 [file nanomaterials-14-00409-s001.zip › nanomaterials-2844865-supplementary.pdf]

Detailed Insight into Photocatalytic Inactivation of Pathogenic Bacteria in the Presence of Visible-Light-Active Multicomponent Photocatalysts

Magda Kozak [1,*], Paweł Mazierski [1], Joanna Żebrowska [2], Tomasz Klimczuk [3], Wojciech Lisowski [4], Andrzej M. Żak [5], Piotr M. Skowron [2] and Adriana Zaleska-Medynska [1,*]

[1] Department of Environmental Technology, Faculty of Chemistry, University of Gdansk, 80-308 Gdansk, Poland; pawel.mazierski@ug.edu.pl
[2] Department of Molecular Biotechnology, Faculty of Chemistry, University of Gdansk, 80-308 Gdansk, Poland; joanna.zebrowska@ug.edu.pl (J.Ż.); piotr.skowron@ug.edu.pl (P.M.S.)
[3] Faculty of Applied Physics and Mathematics, Gdansk University of Technology, 80-233 Gdansk, Poland; tomasz.klimczuk@pg.edu.pl
[4] Institute of Physical Chemistry, Polish Academy of Sciences, 01-224 Warsaw, Poland; wlisowski@ichf.edu.pl
[5] Faculty of Chemistry, Wroclaw University of Science and Technology, 50-370 Wroclaw, Poland;
andrzej.zak@pwr.edu.pl
* Correspondence: magda.kozak@ug.edu.pl (M.K.); adriana.zaleska-medynska@ug.edu.pl (A.Z.-M.);
Tel.: +48-58-523-51-59 (M.K.)

SUPPLEMENTARY MATERIALS

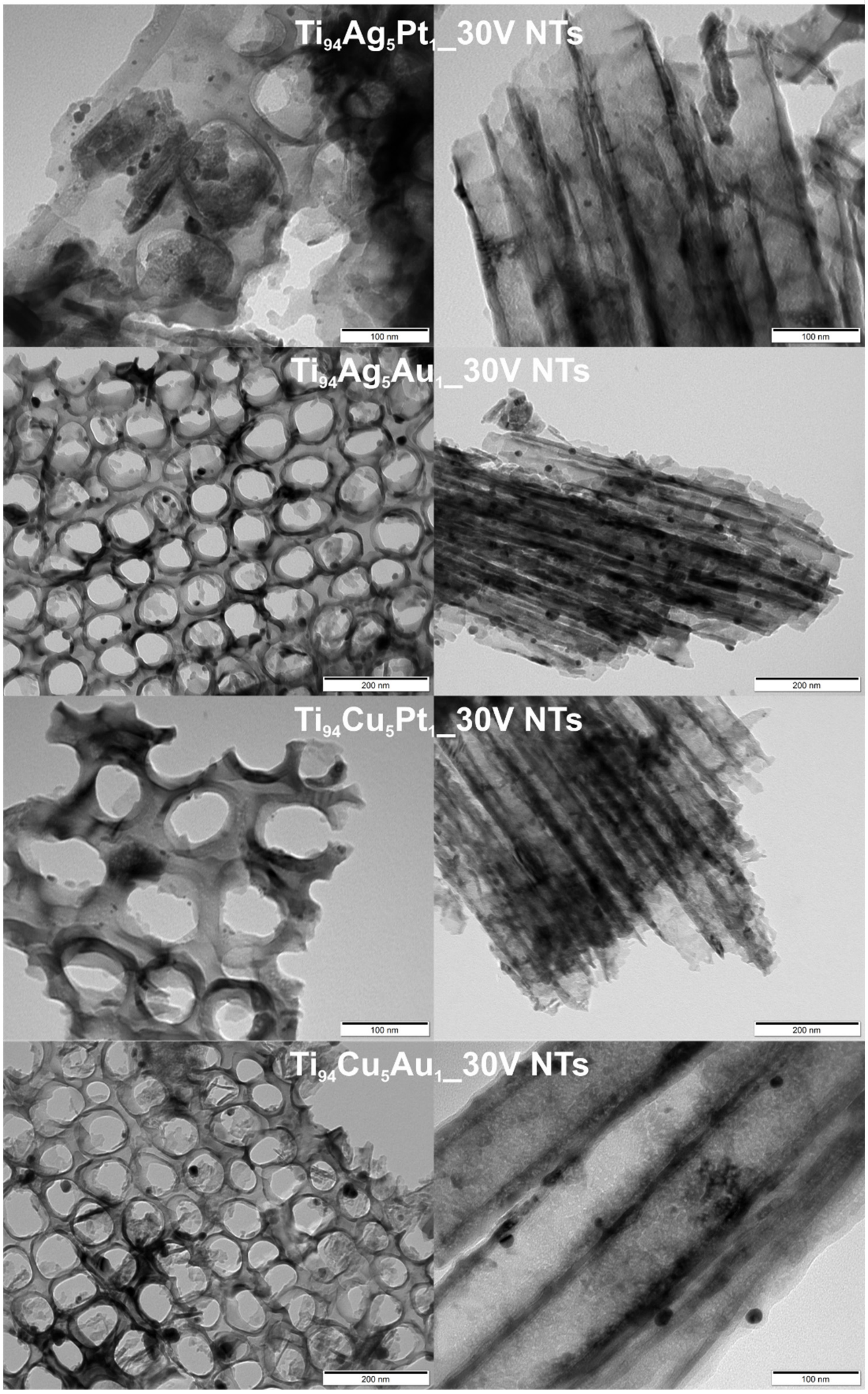

Figure S1. TEM images of modified NT layers prepared from Ti alloys.

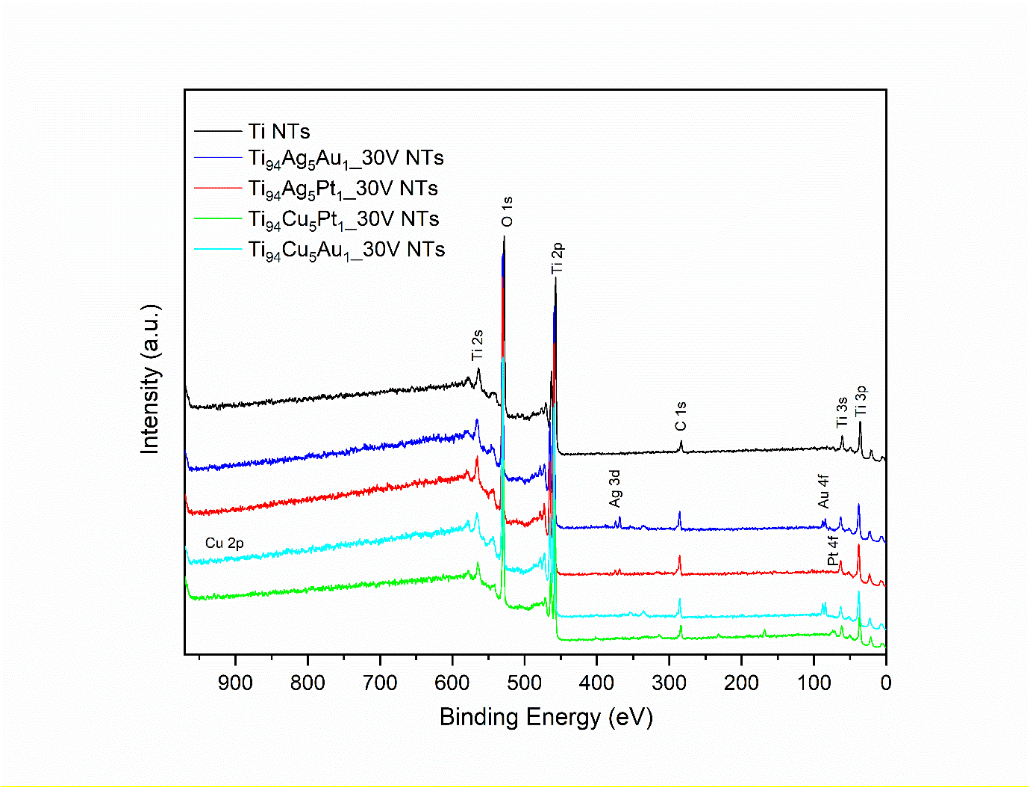

Figure S2. Survey XPS spectra identified on Ti NTs and various Ti-alloy sheets.

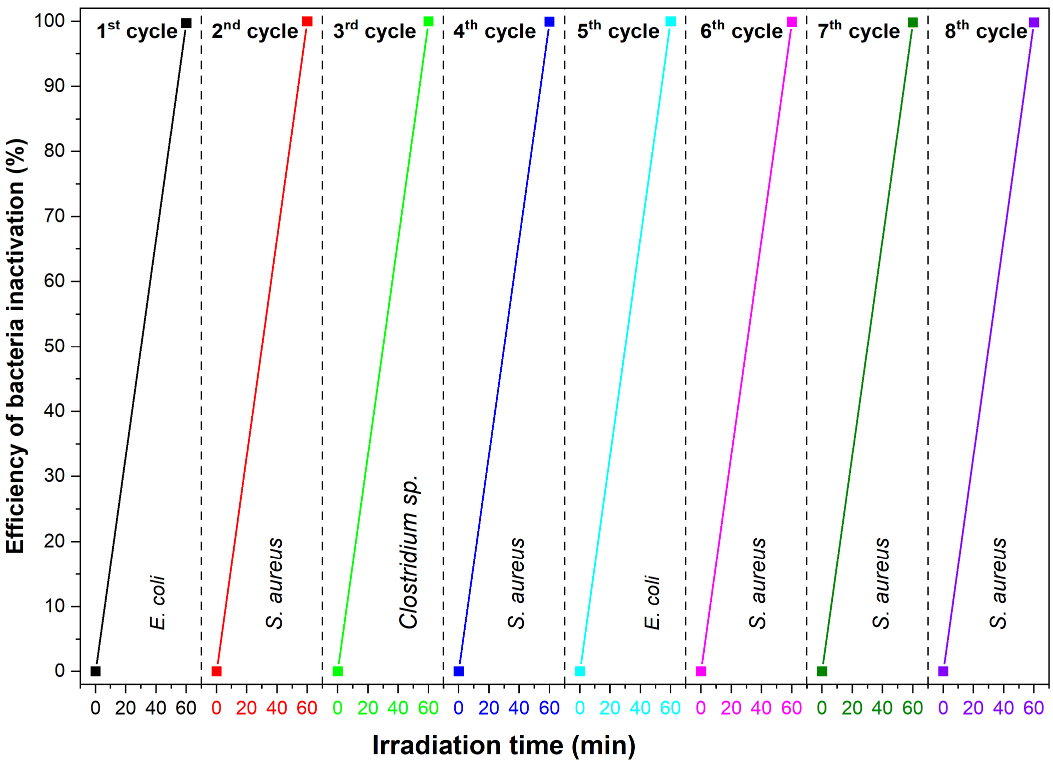

Figure S3. Photocatalysis processes performed in the presence of $Ti_{94}Ag_5Au_1$ NTs sample in 8 reuse cycles.

Table S1. Characteristics of bacteria used in the experiments

Type of bacteria	Characteristics
E. coli	A gram-negative, anaerobic bacteria that contributes to the generation of vitamins B and K62 in the bacterial flora of mammals' intestines. occurs in soil and water, making it a sign that the provided media has been contaminated. *E. coli* infections in people can cause sepsis, nosocomial pneumonia abscesses in organs, and disorders of the urinary and digestive systems [1].
S. aureus	A gram-positive bacteria that can be found in the nose and on the skin. *S.aureus* can show resistance to many antibiotics, which often makes it difficult to treat. Some strains of *S.aureus* can be resistant to standard antibiotics such as penicillin and methicillin, which is known as methicillin-resistant *Staphylococcus aureus* (MRSA) [2]. The polysaccharide capsule plays a vital function in the process of epithelial colonization. The bacterium produces cytolytic toxins and enzymes that aid in its spread and cause tissue destruction in the afflicted host. It is a bacteria that can live in both aerobic and anaerobic conditions. *S.aureus* used in the study is MSSA (methicillin-susceptible *Staphylococcus aureus*) which is sensitive to antibiotics however it can cause serious infections like blood poisoning and toxic shock syndrome where it gets into the bloodstream causing MSSA bacteremia. It is most common not only in healthcare facilities but in a majority of it is prevalent outside hospitals [3]. Differences between MRSA and MSSA are related to the presence or absence of specific antibiotic-resistance genes in MRSA. These genes encode proteins that provide the bacteria with resistance to methicillin and other β-lactam antibiotics. However, the bacterial structure and structure of MRSA and MSSA are themselves similar [4]. However, it is worth noting that the strain used in the study was isolated from a clinical sample from a hospital.
K. oxytoca	A gram-negative bacteria which represents high pathogenicity. It generates beta lactamase, resulting in penicillin and ampicillin resistance. It can be obtained through animals or insects. It is commonly seen in the intestines, skin, and nasopharynx, although it may additionally be found in other regions of the body [5]
Clostridium sp.	A gram-positive bacteria that is rod-shaped, anaerobic and produces oval, lower spores. It is usually found in soil [6].

Table S2. The concentration of CO_2 (μmol/L) varied in the reactor's headspace during the photocatalytic inactivation of bacteria, depending on the specific process utilized

Reaction time (min)	The concentration of CO_2 (μmol/L) from the headspace of the photoreactor depending on the type of process		
	Dark process – lack of photocatalyst, lack of light	Photolysis – lack of photocatalyst, presence of light λ > 420 nm	Photocatalysis – the presence of a photocatalyst and light λ > 420 nm
0	8	6	8
120	17	24	32
240	24	44	53

References

[1] J. Y. Lim, J. Yoon, and C. J. Hovde, "A brief overview of Escherichia coli O157:H7 and its plasmid O157.," *J Microbiol Biotechnol*, vol. 20, no. 1, pp. 5–14, Jan. 2010.

[2] I. R. Cooper, "Introduction to biomaterials and medical device-associated infections," in *Biomaterials and Medical Device - Associated Infections*, Elsevier, 2015, pp. 3–17. doi: 10.1533/9780857097224.1.3.

[3] "https://www.healthline.com/health/mssa."

[4] J. Camacho-Cruz *et al.*, "Differences Between Methicillin-susceptible Versus Methicillin-resistant Staphylococcus aureus Infections in Pediatrics," *Pediatric Infectious Disease Journal*, vol. 41, no. 1, pp. 12–19, Jan. 2022, doi: 10.1097/INF.0000000000003349.

[5] L. Singh, M. P. Cariappa, and M. Kaur, "Klebsiella oxytoca: An emerging pathogen?," *Med J Armed Forces India*, vol. 72, pp. S59–S61, Dec. 2016, doi: 10.1016/j.mjafi.2016.05.002.

[6] W. TD. Wells CL, *Medical Microbiology. 4th edition.* 1996. Accessed: Apr. 25, 2023. [Online]. Available: https://www.ncbi.nlm.nih.gov/books/NBK8219/
